# Clinical Efficiency and Acceptability of EMDR and MOSAIC Therapy for PTSD

**DOI:** 10.3390/healthcare11152226

**Published:** 2023-08-07

**Authors:** Deborah Flatot-Blin, Arnaud Rey, Flavie Derynck, Olivier Fossard, Stephanie Khalfa

**Affiliations:** 1 Laboratoire de Psychologie Cognitive, CNRS, Aix Marseille University, 13003 Marseille, France; 2 Laboratoire de Neurosciences Cognitives, CNRS, Aix Marseille University, 13003 Marseille, France; 3 Assistance Publique des Hôpitaux de Marseille, 13005 Marseille, France; 4 Institute of Language and Communication and the Brain, CNRS, Aix Marseille University, 13604 Aix-en-Provence, France; 5 Centre Hospitalier de Montfavet, 84140 Avignon, France; olivier.fossard@ch-montfavet.fr

**Keywords:** PTSD, EMDR, MOSAIC therapy, ABS, body sensation, pleasantness

## Abstract

Eye movement desensitization and reprocessing (EMDR) therapy is one of the therapies recommended by the World Health Organization (2013) to treat posttraumatic stress disorder (PTSD). Although efficient, repeated exposure to the traumatic memory may reduce its acceptability to patients. The therapy “eye movement and alternate stimulation for brain integration” (MOSAIC in French) was developed to improve acceptability and reduce pain by drawing on the patient’s internal resources. MOSAIC therapy focuses on the body sensations that the patient wants to experience and avoids having to relive the traumatic memories. This observational study aimed to compare the clinical efficiency of EMDR and MOSAIC therapy for PTSD and to measure the well-being generated by both therapies. Twenty-six PTSD patients (17 females and 9 males, mean age 37.01 years, *SD* = 13.06) received treatment by psychiatrists and/or psychologists trained with EMDR or MOSAIC therapy. Both patient groups achieved a significant decrease in PTSD symptoms as measured with the PCL-5. However, fewer sessions were required with the MOSAIC therapy than with EMDR therapy. As expected, the level of well-being experienced by the patient during the therapy, assessed using the Lickert scale, was higher with MOSAIC than with EMDR therapy from the first session. These findings provide the first evidence of the efficacy of MOSAIC therapy treatment, which now needs to be corroborated in a larger randomized clinical trial.

## 1. Clinical Efficiency and Acceptability of EMDR and MOSAIC Therapy for PTSD

Posttraumatic stress disorder (PTSD) is usually considered to develop one month after exposure to a stressful event involving death, risk of death, impairment to oneself or a loved one, or witnessing (DSM-5; [1]). Exposure to the traumatic stressor can be direct or indirect. Symptoms of PTSD are in four categories: (1) intrusions, (2) avoidance, (3) altered cognition and mood, and (4) altered arousal and responsiveness (DSM-5; [1]). PTSD is a major public health problem, with a lifetime prevalence of 8% to 11% in the general population [2]. In addition, 80% of PTSD patients have comorbid disorders such as anxiety, depression, and addictive behaviors [3,4].

For the psychotherapeutic treatment of PTSD, the World Health Organization (WHO) [5] recommends two therapies: cognitive-behavioral therapy (CBT) and eye movement desensitization and reprocessing (EMDR) therapy. CBT includes prolonged exposure therapy (PE), trauma-focused CBT (TF-CBT), and cognitive processing therapy (CPT), among the more evidence-based psychotherapies for PTSD [6]. These therapies are based on the patient’s prolonged exposure to the traumatic event [7]. During the exposure phase, patients must confront traumatic memories and expose themselves to traumatic stimuli to achieve habituation or extinction [7,8]. PE therapy achieves results on PTSD symptoms in 8 to 15 sessions [7] and in 8 to 25 sessions with TF-CBT [9]. For CPT, the protocol consists of 12 sessions, working on the negative beliefs associated with the traumatic memory before focusing on exposure to the traumatic script [10]. These therapies change inappropriate thoughts until the conditioned reaction is extinguished [11]. Habituation mechanisms allow decreasing emotional responses to traumatic events.

In EMDR therapy, a standardized eight-step protocol [12], alternating bilateral stimulations (ABS) are executed while the patient recalls the cognitive, emotional, and physical aspects of the traumatic experience. ABS consists of horizontal eye movements or auditory or tactile stimuli alternately presented at the right and left sides of the body [13,14,15]. This reactivation of traumatic memory associated with ABS allows for a rapid decrease in PTSD-related emotional responses and, thus, a decrease in PTSD symptoms [16]. Seven of eight randomized controlled trials reported that the rate of PTSD remission in patients with single-event PSTD undergoing EMDR therapy was 77% to 90% after three to eight 90-min therapy sessions [13,16]. Various metanalyses compared the efficiency of TF-CBT and EMDR therapy in treating PTSD and did not report significant differences between these therapeutic approaches [17,18].

A key clinical feature of CBT and EMDR therapy is repeated exposure to the trauma memory, which is associated with a risk of dropping out of therapy for up to 30% of patients [17,19]. In 2021, Varker et al. [20] conducted a meta-analysis of patient dropout during recommended exposure therapies and found average rates of 14.9% for EMDR therapy, 18% for CBT, 28.7% for prolonged exposure, and 34% for CPT. This dropout is a critical problem for the treatment of PTSD, and it is frequently associated with the strong abreactions patients may have when remembering the traumatic situation [20]. Additionally, exposure to the patient’s history and abreactions increases therapists’ risk of vicarious trauma as defined in the DSM-5 (2013) and/or compassion fatigue [21].

To protect both patients and therapists from abreactions, dissociation, therapy discontinuation, and vicarious trauma, other therapies have been used for PTSD (e.g., interpersonal therapy, mindfulness, neurofeedback) [22,23]. However, few clinical studies have validated the efficacy of these therapies for PTSD, and early results have instead shown less efficacy on PTSD symptoms [24] and non-inferiority or equivalent efficacy than for trauma memory exposure therapies [25]. Despite the lower efficacy than that for recommended psychotherapies for PTSD, the dropout rate was significantly lower, and acceptability was higher [23]. Acceptability is described as the patient’s judgment of whether the treatment is appropriate for the problem and whether it is perceived as tolerable, fair, and reasonable for the patient [26]. Thus, acceptability is the proportion of patients who continue treatment to completion rather than discontinue due to adverse effects of the therapy [27].

To avoid traumatic memory exposure for better acceptability and less dropout while maintaining the same high level of efficiency as therapies with trauma exposure, we considered a recent therapy developed for PTSD, the MOSAIC therapy (French acronym for *Mouvements Oculaires et Stimulations Alternées pour l’Intégration Cérébrale*; i.e., eye movement and alternate stimulation for brain integration [28,29]. MOSAIC therapy is based on solution-oriented and experiential therapy [30,31]. It mainly focuses on the individual’s resources and solutions and on what patients want to experience instead of what they are experiencing because of their PTSD [29].

MOSAIC therapy is based on a four-step protocol: (1) an initial interview, (2) a reconnection loop, (3) an exchange concerning the more general benefits the patient can derive from the treatment, and (4) a debriefing. First, the initial interview consists of the evocation of the problem, its impact on the patient’s daily life, the benefits the patient derives from not solving the problem, the strategies already in place, the identification of the limiting target (i.e., the most difficult situation/image representing the traumatic event), and the identification of the desired internal sensation (i.e., a body sensation that the patient would prefer to feel when facing the limiting target). Then the desired internal sensation is activated by the patient and associated with sessions of ABS until the desired body sensations are strongly felt. Second, during the reconnection loop, the limiting target is reactivated simultaneously with the desired internal sensation and ABS. Activating the neural network associated with the desired body sensations with the network associated with the limiting target while using ABS reduces the emotional valence of the limiting target. The patient’s feelings are evaluated regularly until the limiting target is no longer disturbing. Third, the therapy’s contribution to the patient’s life consists of a semi-directive exchange to identify and anticipate more generally the benefits the patient will derive from the treatment. The fourth and final step is a debriefing of the session, during which a prescription of sensory or strategic tasks is given to the patient to prolong the effects of the treatment. Therefore, MOSAIC therapy promotes experiential and solution therapy through body sensations. It assumes that resources/solutions already exist within the patient’s brain and body and can be used to transform traumatic memories.

The major advantage of MOSAIC therapy lies in this second phase of the treatment (i.e., the reconnection loop). To explain the critical role of combining the limiting target, the desired internal sensation, and ABS, MOSAIC therapy relies on a neuroscientific model that has been proposed to account for the efficiency of EMDR therapy: the neural stochastic synchronization (NSS) model [28,32]. This model accounts for the effect of ABS on the brain during EMDR therapy through the principle of stochastic resonance [33,34]. Through stochastic resonance, both intra- and inter-regional synchronization of neural activity would be facilitated by adding moderate amounts of random noise [35]. Stochastic resonance would explain how ABS can lead to the emotional desensitization of the traumatic network in EMDR therapy [36]. Indeed, in the NSS model, ABS is considered “noisy” information, without meaning for the brain but with an ability to excite neuronal populations unrelated to the traumatic memory. This noise generated by the ABS amplifies the excitability of neuronal populations initially not activated by the network coding for the traumatic event. By generating this additional neuronal activation, ABS in EMDR therapy elicits the co-activation of these neuronal populations with the neural network coding for the traumatic memory. This produces synchronization of the traumatic neural network with the “ABS neural network”, which creates and/or reinforces neuronal connections between the two networks via Hebbian learning principles [37]. Emotional desensitization of the traumatic network would then be a by-product of this co-activation of the two networks (i.e., the traumatic one and the one generated by ABS). In this way, the traumatic memory would reconsolidate in a memory enriched with new non-traumatic information resulting in relieving this traumatic memory from the weight of its emotions [38]. Note that the NSS model may provide a neural explanation of ABS functioning in EMDR therapy where psychological theories, like the working memory hypothesis, are more often proposed [39,40,41].

MOSAIC therapy was developed from the NSS model, which explains the functioning of ABS in EMDR therapy. It also applies inverse reasoning to EMDR therapy by starting from the desired sensation and not from the traumatic memory to be desensitized. This therapy focuses directly on the patient’s resources and body sensations and amplifies them through ABS. When this desired and chosen interoceptive network is enlarged and “strong” enough with the addition of activated neurons from the ABS, the limiting target can be recalled simultaneously to the desired internal sensations and ABS. This part of the protocol is called the “reconnection loop” that allows the co-activation of the two neural networks (i.e., the desired internal sensation with the ABS network and the limiting target network). In this way, the neuronal networks synchronize, and the traumatic network reconsolidates with the desired interoceptive network, likely leading to a deactivation of the amygdala response [28,29]. If the patient expresses suffering when the two networks meet, the traumatic memory is immediately stopped, and the desired internal sensation is again reinforced with the ABS until the next meeting.

Therefore, MOSAIC therapy proposes a change in therapeutic strategy relative to EMDR therapy. In EMDR therapy, the traumatic memory is activated, and ABS helps to gradually decrease the associated negative emotions, thus leading to a reconsolidation of the desensitized traumatic memory trace. But the desensitization process can be long and painful, leading frequently to intense negative emotions. MOSAIC therapy allows the patient to experience and activate solutions directly by reliving desired internal body sensations. Once this virtuous network of the desired internal sensation has been sufficiently activated, it is coupled with the cognitive recall of the traumatic neural network with ABS. In this way, the traumatic network reduces its negative emotional valence very quickly and painlessly. Although both therapies predict a similar outcome (i.e., emotional release of the traumatic memory), relief should be easier, less painful, and less prone to dropout with the MOSAIC therapeutic strategy.

The objectives of the present observational study were twofold. First, this study aimed to compare the clinical efficacy of EMDR and MOSAIC therapy on PTSD patients. Clinical efficacy was assessed with the PTSD Checklist for DSM-5 (PCL-5) and the number of therapy sessions required to achieve a significant decrease in PTSD symptoms (resulting in a PCL-5 score below the pathological cut-off for PTSD, i.e., 33) [42,43]. Second, it aimed to compare the acceptability of MOSAIC and EMDR therapy by measuring patients’ well-being during therapy sessions. Because EMDR and MOSAIC therapies each allow desensitization of the traumatic memory, we expect a similar clinical efficiency for both therapies. However, we expect well-being to be greater with MOSAIC than with EMDR therapy because patients are less exposed to the traumatic memory.

## 2. Materials and Methods

### 2.1. Participants

This observational study was conducted with patients from two French hospitals. Clinical files for 26 patients with PTSD were selected from the Psychotraumatic Unit in La Conception Hospital in Marseille and Montfavet Hospital in Avignon (France). To quantify PTSD symptoms, the PCL-5 questionnaire was completed by each patient. Patients diagnosed by a psychiatrist as having PTSD (following DSM-5 criteriaand a score higher than 33 at the PCL-5) were included in the study. We excluded patients with complex PTSD (i.e., repeatedly experienced traumatic events) [44] and those with addictive disorders or neurological or psychiatric problems, except for those with anxiety and depressive disorders, if their occurrence was related to PTSD.

Patients received EMDR or MOSAIC therapy from trained psychologists from the two hospitals. These professionals have all been trained in EMDR or MOSAIC therapy with regular individual or group supervision from certified supervisors in either MOSAIC or EMDR therapy. The two groups were matched as closely as possible on age and socio-cultural level. Socio-demographic characteristics and psychometric scores for all patients are described in Table 1. The number of males and females was equal in the MOSAIC group but unbalanced in the EMDR group, which included more females. The traumatic events could be assault (*n* = 11 patients), road accidents (*n* = 8), human-made disasters (*n* = 3), bereavement (*n* = 1), and witnessing with a risk of death (*n* = 1). Several types of medication taken in parallel with the therapy included anxiolytics (*n* = 4 patients), antidepressants (*n* = 9), and neuroleptics (*n* = 1); the remaining 12 patients were not taking any medication. The matching of the two groups on these different dimensions was therefore favored over randomization, as is the case in an RCT. The objective of this observational study is to provide a first test of our hypotheses concerning the MOSAIC therapy, which, if successful, will allow us to obtain funding to carry out an RCT study.

### 2.2. Psychometric Scales

PTSD symptoms were quantified with the PCL-5 and the medical and clinical interview with the psychiatrist. The PCL-5 scale includes the four diagnostic criteria of PTSD documented in the DSM-5: reliving the traumatic event, cognitive and mood alteration, neurovegetative hyperactivation, and avoidance. Patients completed the PCL-5 before and after EMDR or MOSAIC therapy. The scale contains 20 items, and scores range from 0 (not at all) to 4 (extremely) in severity. The PCL-5 score, therefore, varies from 0 to 80, and the pathological cut-off for PTSD is 33 [43]. Cronbach’s alpha analysis showed that the PCL-5 scores of the patient group had good internal consistency (α = 0.90), demonstrating that PTSD symptoms are homogeneous across the patient sample as in other studies [43].

After each therapy session, the painfulness and pleasantness experienced during the session were briefly assessed. Two 10-point Likert scales were used to test for the pleasantness and painfulness of the sessions. The first scale ranged from 0 (not pleasant) to 10 (very pleasant). The second ranged from 0 (not painful) to 10 (very painful).

### 2.3. Procedure

Patients were received at the Post Trauma Consultation Unit from each hospital. They were diagnosed with PTSD by psychiatrists, and their symptoms were quantified with the PCL-5. Following this first evaluation, patients were included in the service’s care program and assigned to a psychologist trained in EMDR or MOSAIC therapy. Five therapists performed the EMDR therapy and two the MOSAIC therapy.

The treatment framework was described to patients, and the therapy was explained. Then, the first psychotherapeutic session was conducted. Each patient was seen weekly or every two weeks. Each session lasted about one hour. At the end of each session, the patient completed the pleasantness and painfulness scales. We retained a maximum of 10 sessions to compare the therapeutic effect of EMDR and MOSAIC therapy. Some patients reached a sufficient reduction of symptoms to complete the psychotherapeutic treatment, and others had to continue the treatment after the tenth session to reach a sufficient reduction in symptoms. A detailed description of each patient’s anonymized individual data is available here (modified and last accessed on 15 May 2023) [45]: https://osf.io/x8gu4/?view_only=217f946f888049a9a3cef4242fae6491.

### 2.4. Statistical Analysis

Statistical analyses were performed using Sigmaplot 14.5 and JASP 0.16.0.0 software. Because each patient had a different number of sessions, only the scores obtained after the first and last sessions were compared. Data are described with mean ± standard deviation (SD). To test the clinical efficiency of MOSAIC vs. EMDR therapy for PTSD, we used a two-factor ANOVA for PCL-5 scores, with therapy (i.e., EMDR vs. MOSAIC) as a between-group factor and session (first vs. last session) as a repeated within-participant factor. A two-sample *t*-test was used to compare the number of EMDR or MOSAIC therapy sessions required to achieve a significant decrease in PTSD symptoms (pre- and post-treatment). Finally, to test the effect of the therapy on the pleasantness and painfulness scores, we used two-factor ANOVA for both scores, with therapy (i.e., EMDR vs. MOSAIC) as a between-group factor and session (first vs. last session) as a repeated within-participant factor. We applied a Bonferroni correction for post-hoc tests when interactions were significant [46]. Table 1 reports, for each group, the total number of sessions, the mean interval between sessions, the delay between the traumatic event and treatment, and the duration of therapy.

## 3. Results

### 3.1. PCL-5

**Figure 1:**
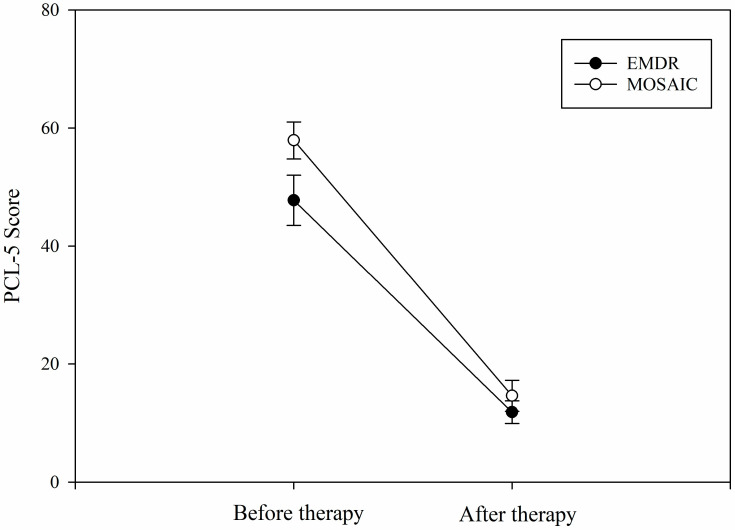
PCL-5 scores before and after MOSAIC and EMDR therapy. Note. Error bars are standard errors. EMDR: eye movement desensitization and reprocessing; MOSAIC: eye movement and alternate stimulation for brain integration.

We found a main effect of session (*F*(1, 24) = 162.45, mean squared error (*MSe*) = 20,441.56, *η*^2^*_p_* = 0.734, *p* < 0.001; Figure 1) on PCL-5 scores. All patients showed a similar reduction in PTSD symptoms and PCL-5 scores with both therapies in the last session (mean score = 13.24) compared to the first session (mean score 51.35). Indeed, we found no main effect of therapy nor any interaction between therapy and session (all Fs < 1). For EMDR therapy, the mean PCL-5 scores were 44.77 and 11.85 before and after the therapy, respectively. For MOSAIC therapy, the mean PCL-5 scores were 57.92 and 14.62, respectively.

### 3.2. Number of Sessions

The mean total number of therapeutic sessions was lower with MOSAIC than with EMDR therapy (3.1 ± 1.7 vs. 5.4 ± 2.4) (*t*(24) = 2.88, *p* = 0.008).

### 3.3. Pleasantness Scale

**Figure 2:**
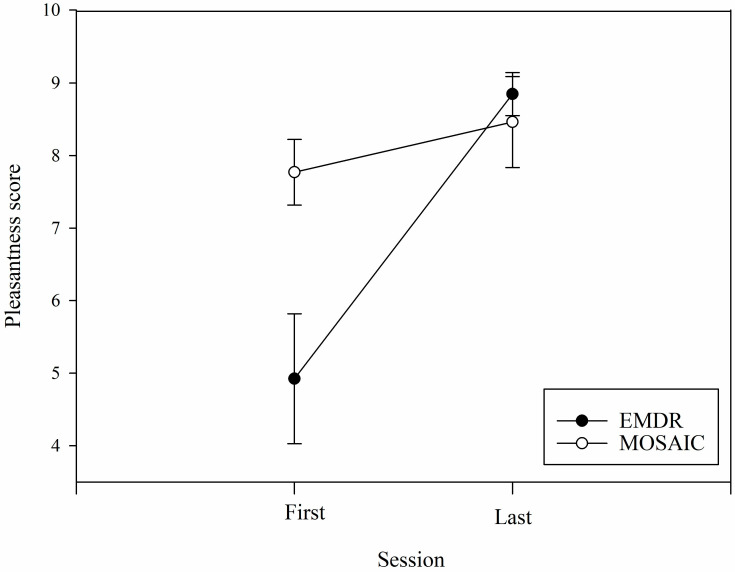
Evolution of the mean pleasantness scores for the two therapies (i.e., EMDR and MOSAIC). Note. Data are mean and standard error.

A primary effect of the session related to the pleasantness scale (*F*(1, 24) = 15.15, *MSe* = 71.56, *p* < 0.001, *η*^2^*_p_* = 0.387; Figure 2), the last session being more pleasant than the first (mean score 7.5 vs. 6.35). We found a main effect of therapy (*F*(1, 24) = 4.17, *MSe* = 20.94, *p* = 0.05, *η*^2^*_p_* = 0.148), indicating that MOSAIC therapy produced more pleasantness than EMDR therapy (mean score 8.28 vs. 6.93). We also found a significant session-by-therapy interaction (*F*(1, 24) = 6.84, *MSe* = 32.33, *η*^2^*_p_* = 0.222, *p* = 0.02), indicating that the increase in pleasantness from the first to the last session was higher with EMDR than MOSAIC therapy (*t*(24) = 3.29, *p* < 0.004).

### 3.4. Painfulness Scale

**Figure 3:**
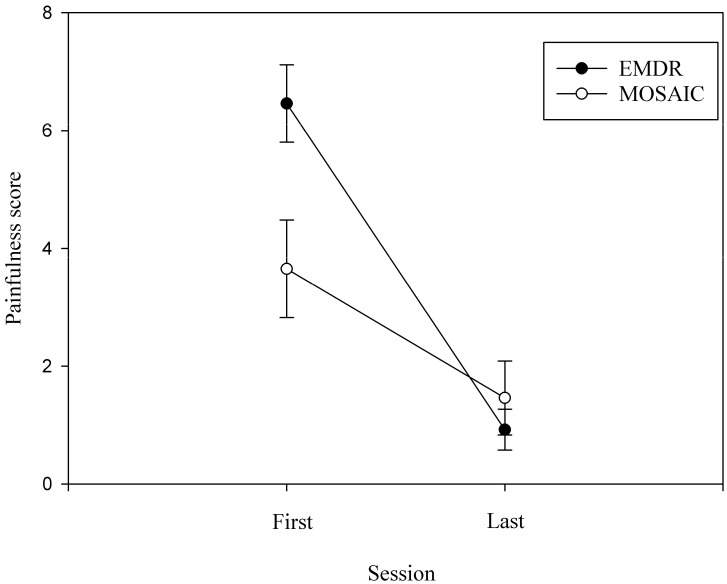
Evolution of the mean painfulness scores for the two therapies (i.e., EMDR and MOSAIC). Note. Data are mean and standard error.

We determined a primary effect of the session on the painfulness scale (*F*(1, 24) = 37.81, *MSe* = 198.12, *p* < 0.001, *η*^2^*_p_* = 0.612; Figure 3), the last session being less painful than the first (mean score 1.19 vs. 5.06). The effect of therapy was not significant (*F*(1, 24) = 3.27, *MSe* = 17.89, *p* = 0.083, *η*^2^*_p_* = 0.12). We also found a significant session-by-therapy interaction (*F*(1, 24) = 6.628, *MSe* = 34.74, *p* = 0.02, *η*^2^*_p_* = 0.216), indicating that patients had a stronger decrease in painfulness with EMDR than MOSAIC therapy (*t*(24) = −3.09, *p* < 0.006).

## 4. Discussion

In the present observational study, we tested the clinical efficacy and acceptability of EMDR and MOSAIC therapy for PTSD. For both therapies, the post-treatment PCL-5 scores were significantly lower than the pre-treatment scores and were even lower than the pathological cut-off for PTSD (PCL-5 ≥ 33) [43]. In addition, the number of therapy sessions required to achieve a significant decrease in PTSD symptoms was lower for MOSAIC therapy than for EMDR therapy. Finally, patients experienced greater comfort with MOSAIC than EMDR therapy (i.e., more pleasantness and less pain).

Our results show a comparable significant decrease in PTSD symptoms (flashbacks, mood alteration, and hypervigilance, reported during the PCL-5 evaluation) with both therapies. For all patients, the PCL-5 score was below the pathological threshold of 38/80 after the therapeutic intervention. According to numerous studies and meta-analyses, EMDR has one of the highest efficacies in reducing PTSD symptoms, with sustained effects at 1 to 4 months [18]. Therefore, our data suggest that MOSAIC therapy may reach a similar level of efficacy as EMDR therapy.

Our results also indicate that patients with PTSD required fewer sessions of MOSAIC than EMDR therapy to achieve a significant decrease in PTSD symptoms. The number of sessions to reach such a decrease seems consistent with previous studies. Shapiro [16] noted that completing 3 to 8 sessions of EMDR therapy resulted in symptom reduction (pre- and post-treatment) in 84% of patients diagnosed with simple PTSD. This difference in the number of sessions was not expected and could be related to the therapeutic strategy of MOSAIC therapy. Because of its solution-oriented approach, MOSAIC therapy produces a faster reduction of negative emotions by activating the desired internal sensation before the limiting target [28,29]. According to the neural stochastic synchronization (NSS) model [28,32,38], this therapeutic strategy may indeed produce a faster decrease of the emotional valence related to the limiting target because of the reconnection loop between the limiting target and the desired internal sensation and the absence of prolonged exposure to the trauma memory.

We also found greater pleasantness scores for MOSAIC than for EMDR therapy during the first therapeutic session. This finding is consistent with previous studies indicating that solutionist and sensitive therapies produce more comfort and are more acceptable than trauma-focused therapy [23]. With MOSAIC therapy, from the very first session, patients experienced pleasantness because they initially focused on a desired internal sensation in their body [29]. Recent studies have found that focusing on body sensations helps patients feel calmer and stronger and reduces PTSD symptoms [47]. Focusing on body sensations also seems interesting because awareness of one’s sensations seems to be negatively correlated with PTSD symptoms [48], as in the case of dissociations, in which the patients are no longer connected to their bodies. Therefore, focusing on pleasant body sensations could lead to less dissociative symptoms.

Our results also indicate a significant difference in the painfulness felt by patients between EMDR and MOSAIC therapy during the first therapeutic session, with EMDR therapy producing more painfulness than MOSAIC therapy. The risk of experiencing painfulness and abreactions is increased with EMDR therapy and may lead to a higher dropout [5]. However, at the end of the therapy, the pain level was low for both therapies, as was the level of pleasantness, which reached similar levels. Thus, at the end of treatment, EMDR and MOSAIC therapies were equally acceptable for all patients.

The present set of results is consistent with the predictions from the NSS model, which explains why the primary benefits of MOSAIC therapy relate to the upstream activation of the desired internal sensation. When the desired internal sensation is felt, a neural network of pleasant body sensations is activated. The neural network of the desired internal sensation is then associated with the ABS, which leads to a larger and stronger well-being network of body sensations. During the reconnection loop, the well-being network and the traumatic network are associated. This co-activation generally leads to a very fast decrease in the negative emotional strength of the traumatic network.

## 5. Limitations

This study is the first to investigate the clinical efficiency of EMDR therapy compared to MOSAIC therapy. It is an observational study using routine care data, with a relatively small number of patients in the two treatment groups and no randomization. Larger sample sizes are needed to confirm our results. Second, although all therapists are trained in EMDR or MOSAIC therapies and receive regular supervision, the number of therapists in each group was relatively small. Thus, demonstration of treatment fidelity through a clinical trial [49] could not be achieved in this observational study because (a) MOSAIC therapy is new and (b) the number of trained therapists is still limited. It would, therefore, be helpful to increase this number to avoid any therapist effects. Third, some of our results may be due to confounding factors, such as the use of drug treatments, the type of trauma experienced by the patients, and the presence of comorbidities not evaluated in this preliminary study. The results of this preliminary non-interventional study, therefore, need to be confirmed by a randomized controlled trial with larger groups of patients to overcome these limitations. Finally, this study did not provide information on the long-term effects of MOSAIC therapy, or the comfort felt by the therapists. Future studies are also needed to address this question.

## 6. Conclusions

This observational study reports a similar efficacy of EMDR and MOSAIC therapy for PTSD. It also suggests superior comfort with MOSAIC than with EMDR therapy, at least in the early phases of the treatment, so MOSAIC may be more acceptable than EMDR therapy for patients with PTSD (and probably even for therapists). This greater comfort may explain why we also found fewer MOSAIC than EMDR sessions reached a significant decrease in PTSD symptoms. Altogether, greater comfort and faster positive outcomes should reduce the dropout risk. Thus, the results of this observational study are encouraging for the future of psychotherapeutic treatments for PTSD. MOSAIC therapy is a novel, non-trauma-focused therapy that could significantly improve PTSD symptoms without painful exposure to the traumatic memory, thus limiting the risk of therapy discontinuation and vicarious trauma or therapist compassion fatigue. MOSAIC therapy could expand the range of treatment options for PTSD patients. A further randomized controlled trial is needed to confirm and extend these initial encouraging results for PTSD treatment.

## Figures and Tables

**Table 1:** Socio-demographic characteristics of patients receiving EMDR or MOSAIC therapy (gender, age, education level) and psychometric scores for each therapy (number of sessions, delay between the traumatic event and the therapy, duration of the therapy).

	EMDR	MOSAIC	*p*-Value
Socio-demographic characteristics			
Male	*n* = 6	*n* = 3	
Female	*n* = 7	*n* = 10	
Age (years)	38.2 (12)	37.4 (13.7)	ns
Education level (years)	12.9 (2.3)	13.5 (2)	ns
Psychometric scores			
Number of sessions	3.1 (1.7)	5.4 (2.4)	0.008
Delay between traumatic event and therapy (days)	533.3 (511)	924.7 (1435)	ns
Duration of therapy (days)	17.3 (10.8)	51.6 (35.7)	0.005

Note. Data are mean and standard errors (in brackets). Two-sample *t*-tests were used to compare the groups, and *p*-values are provided for each mean comparison. EMDR: eye movement desensitization and reprocessing; MOSAIC: eye movement and alternate stimulation for brain integration. “ns” for not significant.

## Data Availability

The data described in this article are available on Open Science Framework at https://osf.io/x8gu4/?view_only=217f946f888049a9a3cef4242fae6491 (modified and last accessed on 15 May 2023).
